# Home, head direction stability, and grid cell distortion

**DOI:** 10.1152/jn.00518.2019

**Published:** 2020-02-26

**Authors:** Juan Ignacio Sanguinetti-Scheck, Michael Brecht

**Affiliations:** ^1^Bernstein Center for Computational Neuroscience, Humboldt-Universität zu Berlin, Berlin, Germany; ^2^NeuroCure Cluster of Excellence, Humboldt-Universität zu Berlin, Berlin, Germany

**Keywords:** grid cell, head direction, home, homing, navigation

## Abstract

The home is a unique location in the life of humans and animals. In rats, home presents itself as a multicompartmental space that involves integrating navigation through subspaces. Here we embedded the laboratory rat’s home cage in the arena, while recording neurons in the animal’s parasubiculum and medial entorhinal cortex, two brain areas encoding the animal’s location and head direction. We found that head direction signals were unaffected by home cage presence or translocation. Head direction cells remain globally stable and have similar properties inside and outside the embedded home. We did not observe egocentric bearing encoding of the home cage. However, grid cells were distorted in the presence of the home cage. While they did not globally remap, single firing fields were translocated toward the home. These effects appeared to be geometrical in nature rather than a home-specific distortion and were not dependent on explicit behavioral use of the home cage during a hoarding task. Our work suggests that medial entorhinal cortex and parasubiculum do not remap after embedding the home, but local changes in grid cell activity overrepresent the embedded space location and might contribute to navigation in complex environments.

**NEW & NOTEWORTHY** Neural findings in the field of spatial navigation come mostly from an abstract approach that separates the animal from even a minimally biological context. In this article we embed the home cage of the rat in the environment to address some of the complexities of natural navigation. We find no explicit home cage representation. While both head direction cells and grid cells remain globally stable, we find that embedded spaces locally distort grid cells.

## INTRODUCTION

Animals maintain and update a representation of their location in space. Such mapping abilities allow them to navigate their surroundings in search for food, safety, mates, or their kin. In the case of the migratory bar-tailed godwit such navigation can involve a nonstop 11,000 km flight from New Zealand wintering grounds to artic Siberian breeding grounds (Gill et al. 2005) a truly incredible navigational feat. The home is a unique location in the life of humans and animals. Numerous behavioral studies have researched homing in pigeons (Alleva et al. 1975), in bees is returning to the hive (Menzel et al. 2005), in salmon returning to the stream where they were born (Neave 1964), in bats returning to their cave from foraging their favorite tree (Tsoar et al. 2011), and many other species. In rats, the homing site is as a multicompartmental burrow system connected to a complex outside world (Calhoun 1963).

On the other hand, the study of the neural bases of spatial navigation has been a remarkable success story, with the discovery of cells encoding allocentric space. Head direction cells, goal direction cells, and grid cells have been hypothesized to sustain vectorial navigation (Banino et al. 2018; Kubie and Fenton 2009; Sarel et al. 2017; Valerio and Taube 2012).

Head direction cells, present in several brain structures including the anterior dorsal nucleus of the thalamus, the presubiculum, the medial entorhinal cortex (MEC), and the parasubiculum (PaS), have sharp tuning curves in relation the animals orientation in space (Tang et al. 2016; Taube 1995; Taube et al. 1990). In laboratory navigation tasks the accuracy of head direction cells also predicts successful navigation (Valerio and Taube 2012).

Grid cells of the MEC and PaS are known to be active in multiple spatial firing fields that tile the whole environment, forming a periodic hexagonal lattice (Boccara et al. 2010; Fyhn et al. 2004; Hafting et al. 2005; Tang et al. 2016), even though recent work points toward differences in field firing rates as a way for a single cell to encode different environments and local positional information (Diehl et al. 2017; Ismakov et al. 2017; Stensola et al. 2015).

These studies for the most part have been restricted to studying rats and mice in abstract, cue-deprived simple environments. In nature, however, the space inhabited by animals is not minimalistic. It is complex, dynamic, cue ridden, salient, multicompartmental, multiscale, and multipurpose. We are just beginning to study how the nature of the environment influences the properties of grid cells and head direction cells.

Testing how grid cells operate in natural environments requires studies that intervene on the structure of well learnt, familiar environments without disturbing the global environment itself. We find that the home cage of the rat is instrumental for probing effects of biologically meaningful environments. It is a very familiar and rewarding place for the rat from which it performs spontaneous behaviors like navigational trips and pellet hoarding (Maaswinkel and Whishaw 1999; Winter et al. 2018). Safety considerations shape rat exploratory behaviors and laboratory rats naturally organize their behavior around their home cage (Whishaw et al. 2006).

In this paper we assess the neural effects of introducing an embedded space: the laboratory rat’s most relevant local structure, its home cage.

We ask how the introduction or translocation of the home cage is represented by neurons in the MEC and PaS, a structure containing a high proportion of head directional and spatially selective cells, including grid cells and border cells (Boccara et al. 2010), connects selectively to pyramidal patches in layer 2 of the MEC (Tang et al. 2016), and whose head directional activity precedes development of grid cells in the MEC (Langston et al. 2010).

Specifically, we ask the following questions:

1)  How does an embedded home alter head directional encoding?
2)  Are there egocentric home-bearing cells, whose discharge is tuned to the home cage position?
3)  Are grid cells affected by the presence of an embedded space?
4)  Do the saliency and familiarity of the home cage have an effect on grid cells?
5)  Does the explicit use of the home for a behavioral task alter head directional and grid cell signals?

We did not observe home direction cells and found that head direction signals are not affected by the home location. Grid cell signals were locally altered by the home cage location, but the effects appeared to be more geometrical in nature, i.e., related to the addition of walls and change of the internal structure of space, rather than home specific.

## MATERIALS AND METHODS

All experimental procedures were performed according to the German guidelines on animal welfare under the supervision of local ethics committees under permit G0170/15.

### 

#### Subjects.

We obtained data from six male Long Evans rats (~300 g) using chronic extracellular recordings.

#### Tetrode recordings.

Tetrode recordings from PaS and MEC were performed, as recently described (Tang et al. 2016).

Tetrodes were turned from 12.5-μm-diameter nichrome wire (California Fine Wire Company) and gold plated to 250–300 kΩ impedance. To identify tetrodes in the complex anatomy of the PaS and MEC, we stained tetrodes with fluorescent tracers DiI and DiD (ThermoFisher Scientific) before implantation.

Rats were chronically implanted with 32-channel Harlan-8 Drives (Neuralynx) with independently movable tetrodes.

Spiking activity and local field potential were recorded at 32 kHz (Neuralynx; Digital Lynx) or at 32 kHz using the wire free RatLogger-32 from Deuteron Technologies Ltd. All recordings were done freely moving during behavioral tasks. The animal’s location and head direction were automatically tracked at 25 Hz by video tracking with a colored camera using red-blue head-mounted LEDs or red-blue colored plastic targets. After recordings, the animals were transcardially perfused. Spikes were detected and clustered using Kilosort (Pachitariu et al. 2016) and manually curated based on principal component analysis overlap and refractory period violations using the visualization toolbox Phy.

#### Behavioral procedures.

After surgery animals were adapted for 2 wk to a modified home cage with two side doors. Concurrently, the animals were put under food restriction up to achieving 80% of their ad libitum feeding body weight and adapted to foraging small chocolate treats while familiarized with a cue-rich 1×1-m arena under well-lit conditions.

Once the tetrode reached the PaS and MEC (based on the presence of strong theta), we recorded two to eight sessions per day of 12–25 min each, while the animal explored the same familiar arena without removing or disorienting the rat between sessions. In between each session we introduced, or displaced the home cage of the animal or additional control objects (Bottle, Plain Box, and Corridor).

#### Pellet hoarding paradigm.

In a subset of sessions (*n* = 5 rats) we performed hoarding behavioral tests. For these we positioned the home cage in the center of the arena, and instead of randomly dispersing chocolate treats we dispersed standard food pellets outside the rats home cage. Food-deprived rats retrieved these pellets and horded them inside the home cage without any specific training. Rats hoarded up to 80 pellets in 20 min.

#### Hoarding task versus no task.

To dissociate the possible effect of the home location with the effect of the behavioral task, neural recordings were performed comparing No Task behavior. That is to say, that both in absence (open field) or presence of the home, rats were simply randomly foraging for minimal sugary treats. This allowed for a fair behavioral comparison and the necessary occupancy for grid cell analysis.

#### Histology.

After perfusion, the brain was postfixed in Paraformaldehyde 4% for 12–18 h. The brain was then sectioned tangentially using the methods described in (Lauer et al. 2018) and recording sites assigned by histology using immunohistochemistry of calbindin to correctly assign the PaS and MEC recordings.

We did not see significant differences in the populations and pooled cells from PaS and MEC.

#### Analysis of spatial modulation.

The position of the rat was defined as the midpoint between two head-mounted LEDs or colored targets. A running speed threshold (of 5 cm/s) was applied for isolating periods of rest from active movement. Color-coded firing maps were plotted. For these, space was discretized into pixels of 2×2 cm, for which the occupancy *z* of a given pixel *x* was calculated as

$$z(x)=\sum_{t}w(\left|x-x_{t}\right|)\Delta t$$

where *x_t_* is the position of the rat at time *t*, Δ*t* the interframe interval, and *w* a Gaussian smoothing kernel with σ = 5 cm.

Then, the firing rate *r* was calculated as

$$r(x)=\frac{\sum_{i}\left(\left|x-x_{i}\right|\right)}{z}$$

where *x_i_* is the position of the rat when spike *i* was fired. The firing rate of pixels, whose occupancy *z* was less than 20 ms, was considered unreliable and not shown.

For spatial and head directional analysis, both a spatial (>50% spatial coverage) and a firing rate inclusion criterion (>0.5 Hz) were applied. Spatial coverage was defined as the fraction of visited pixels (bins) in the arena to the total pixels.

#### Analysis of spatial information.

For all neurons, we calculated the spatial information rate, *I*, from the spike train and rat trajectory:

$$I=\frac{1}{T}\int r\left(x\right)\log_{2}\frac{r\left(x\right)}{\bar{r}}o\left(x\right)\text{d}x$$

where *r*(*x*) and *o*(*x*) are the firing rate and occupancy as a function of a given pixel *x* in the rate map; *r̅* is the overall mean firing rate of the cell, and *T* is the total duration of a recording session (Skaggs et al. 1993). A cell was determined to have a significant amount of spatial information if the observed spatial information rate exceeded the 95th percentile of a distribution of values of *I* obtained by circular shuffling. Shuffling was performed by a circular time shift of the recorded spike train relative to the rat trajectory by a random time for 1,000 permutations.

#### Analysis of grid cells.

Grid scores were calculated, using publicly available codes from the Derdikman Laboratory’s recent publication (Ismakov et al. 2017), by taking the autocorrelogram, centered on but excluding the central peak. The Pearson correlation of the autocorrelogram with its rotation for 60° and 120° was obtained (on peak rotations) and also for rotations of 30°, 90°, and 150° (off-peak rotations). Gridness was defined as the minimum difference between the on-peak rotations and off-peak rotations.

#### Downsampling of grid cell rate maps for matched speed.

We performed a speed matching control to see whether our grid node shifts persisted with matching speeds between sessions. We performed this speed matching according to (Butler et al. 2019). We divided the map in 10-cm bins; for each of these bins we binned speed into 15 cm/s bins. We counted for each bin in space the amount of time spent by the rat in each speed bin for the open field and home cage sessions. We next downsampled randomly in a bin-specific way to match the session that presented the lowest time for that space and speed bin. We repeated this procedure for each space×speed bin. Finally, to account for random sampling procedure we repeated this procedure 50 times and averaged the rate maps generated.

#### Analysis of head directionality.

Head direction tuning was measured as the eccentricity of the circular distribution of firing rates. For this, firing rate was binned as a function of head direction (*n* = 36 bins). A cell was said to have a significant head direction tuning if the length of the average vector exceeded the 95th percentile of a distribution of average vector lengths calculated from shuffled data and had a Rayleigh vector length >0.3. Data was shuffled by applying a random circular time shift to the recorded spike train for 1,000 permutations.

We studied the head directional properties across subsequent conditions with the presence of the home by analyzing changes in both the angle of the Rayleigh vector or the modulus of the vector.

#### Home direction analysis.

Home direction is calculated as in Fig. 3*A*, as the angle the rat would have to turn its head to face in the direction of the home. In other words, the angle between two vectors defined by the head direction of the animal and a vector between the position of the animal and the position of the home. We applied the same Rayleigh vector analysis to cells in the home direction space and the same cutoff as head direction cells.

#### Multipoint bearing direction analysis.

We performed egocentric bearing analysis in relation to multiple points in the arena. To do this we calculated egocentric bearing Rayleigh vector lengths with respect to a grid of 17×17 (6-cm separation). For each cell we also calculated the maximum vector length (Max VL), the maximal vector length for all the positions of the grid. The position for which the cell has a MaxVL was also considered in this study.

We also pursued the statistical significance of their maximum mean vector length by shuffling the timing of the spikes for each cell 500 times and calculating the maximum vector length for each shuffle. We considered “significantly nonuniform” cells whose maximum vector length was higher than the maximum vector length of 95% of its shuffles.

#### Grid cell sliding window correlation analysis.

Given the overall global stability of grid cells, to compare the population of grid cells in the presence or absence of the home we used a sliding window correlation method as described in Wernle et al. (2018).

For each cell we start with two normalized rate maps of the cell, one for each condition being compared. We choose for each cell a window of size equal to the spacing of the grid cell. This was the window size reported by Wernle et al. (2018).

Starting in one corner of the rate maps, we correlate between both maps the rate of active bins covered by the same window. We assign this local correlation value to the position of the center bin of such window. By sliding the window along all the bins of the rate maps we end up with a local correlation heat map that allows us to dissect local changes in each grid cell resulting from the introduction of the home. In all cases, nonexistent values were removed from the correlations.

To compare the effect of the home in the population we averaged the local correlation heat maps.

#### Classification of cells into functional categories.

Cells were classified as head direction cells, pure grid cells, conjunctive grid cells, and rest cells, based on their grid score, spatial information, and significance of head directionality according to the following criteria:

•  Head direction cells: Rayleigh head direction vector length >0.3 and significant head direction tuning.
•  Pure grid cells: grid score >0.42 and significant spatial information.
•  Conjunctive grid cells: pass criteria for both grid cells and head direction cells.
•  All other cells are considered in the Rest category.

Besides the cell classification, for comparison of different sessions for the same cells we used a stability criterion for cell removal if the *Z*-score of the firing rate for a session goes below −1.5 of the previous sessions.

## RESULTS

**Fig. 1.**
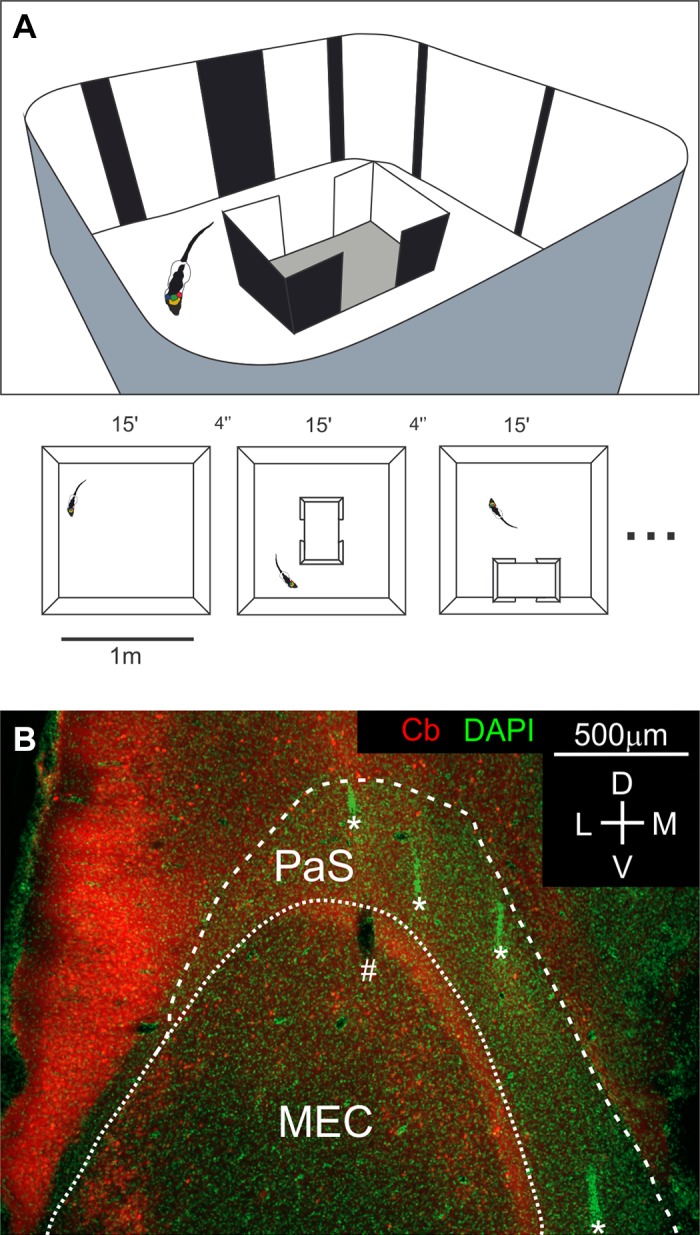
Home paradigm and tetrode recordings. *A*, *top*: schematic of the test environment with the home cage in the center; the animal’s home cage was modified such that not only could the lid be removed, but also two gaps in the sides of the cage could be opened. Walls are covered in complex cues. *Bottom*: we recorded 12- to 25-min sessions without removing the rat from the arena. *B*: histology of tetrode recordings in the medial entorhinal cortex (MEC) and the parasubiculum (PaS). Fluorescence microscopy of a tangential section of layer 2/3 border of the PaS and MEC. Red: calbindin (Cb); green: DAPI staining. The calbindin stripe clearly demarcates the end of the PaS and beginning of MEC. Tetrode tracks are observed as either holes in the section or highly DAPI-fluorescent tracks. Tracks of 4 tetrodes in the PaS are demarcated with asterisks, while a single track in the MEC is demarcated with a hashtag. D, dorsal; L, lateral; M, medial; V, ventral.

In our study we addressed the question how neurons in the rat medial entorhinal cortex and PaS represent complex compartmentalized environments. To this end we recorded from freely moving male Long Evans rats (*n* = 6) using tetrodes. Rats were familiarized for 2 wk with a 1×1-m squared environment with round edges containing a principal cue card and multiple irregularly shaped subcues, on both the walls and the floor of the arena (Fig. 1*A*). During the same period, we housed rats in home cages modified with two side doors to use as an embedded space with which the animal was familiar (Fig. 1*A*).

We recorded sessions of 12–25 min starting with an open field recording and following with sessions where we placed the rat’s home cage in different places in the arena. In order not to disturb the familiarity of the rat with the arena, we did not remove or disorient the rat between sessions. This was facilitated by the use of a wireless logger system for recording which allowed us to test exclusively for the effects of locally altering the internal geometry of the environment. Using tetrodes we recorded cells (*n* = 500) in the PaS, MEC, and medial MEC (Ray et al. 2017) (Fig. 1*B*), which we classified into pure head direction cells (*n* = 90), pure grid cells (*n* = 35), conjunctive grid cells (*n* = 50), and Rest (*n* = 325). We analyzed whether the presence of the embedded space resulted in pure or conjunctive representations of egocentric bearing direction and whether the presence of the home cage affects the encoding of the compartmentalized environment by head direction cells and grid cells.

### 

#### Head direction activity is not affected by the embedded home.

**Fig. 2.**
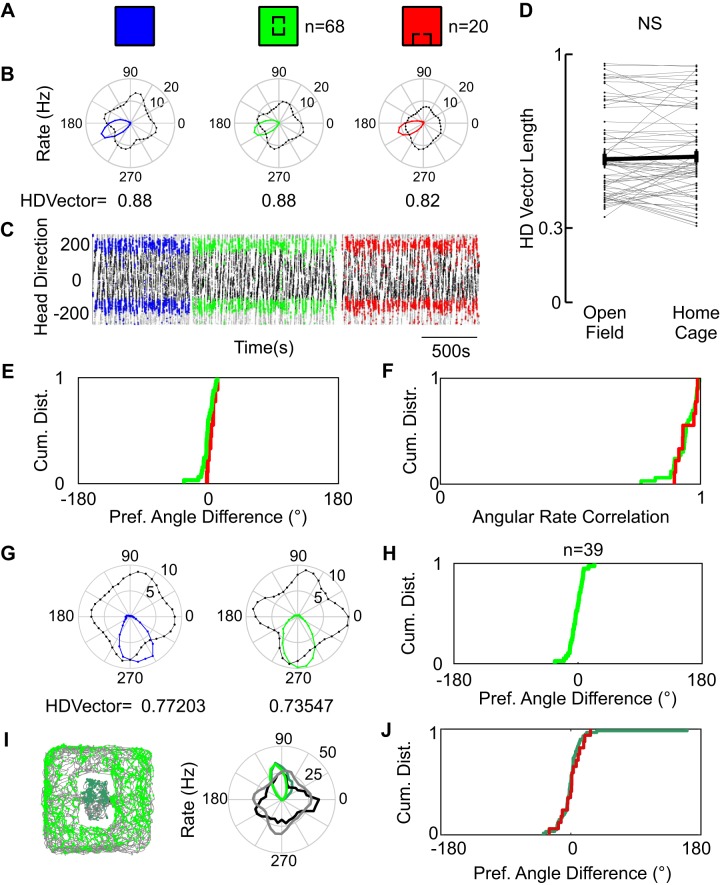
Head direction (HD) discharge is not affected by the embedded home. *A*: schematic of the conditions depicted for a head direction cell, open field (blue), home cage center (green), home cage moved (red). *B*: head direction rate polar plots (rate in corresponding color, angular occupancy in black). *C*: spikes of the head direction cell are clearly preferring a stable head direction. Black and gray, head direction of the animal in time (duplicated for visualization). Spikes are plotted on top in the corresponding RGB color scheme. *D*: head direction Rayleigh vector lengths are not affected by the presence of the home cage. Gray lines show individual cells. Means are depicted in black. Kolmogorov–Smirnov (KS) normality test, *P* = 0.34, *t* test, *P* = 0.96, *N* = 68. *E*: cumulative frequency function of the distributions of differences between preferred angle in the open field and in each home cage condition. Note that the inflection point is at 0 and the very steep slope. KS normality test for angle difference, *P* = 0.6993. *F*: correlation between the angular rates of both home conditions in relation to the open field. Cumulative frequency graph shows that correlations are distributed close to 1. *G*: head direction polar plots of a conjunctive grid cell show a consistent head directionality between sessions in open field (blue) and home center (green) conditions. *H*: cumulative distribution of preferred angle differences in conjunctive grid cells. KS normality test, *P* = 0.26, *N* = 39. *I*: head directional representation inside and outside of the home cage. *Left*: separation of spikes from a head direction cell into inside spikes (dark green) and outside spikes (bright green). *Right*: polar plot of head direction rates inside (dark green) and outside (bright green), and their corresponding head directional occupancy for outside (gray) and inside (black). *J*: cumulative frequency function of the distributions of differences between preferred angle inside and outside of the embedded space. Dark green corresponds to the embedded space in the center. Dark red corresponds to the embedded space rotated and on the side (KS normality test for angle difference, *P* = 0.013, *N* = 62).

As the first step of our analysis of neural responses we assessed how the embedded home affected head direction signals (Fig. 2). We recorded head direction cells (*n* = 68) in the PaS/MEC with the presence of the home in the center of the environment or the home rotated and translated to the edge of the environment (*n* = 20; Fig. 2*A*) and found that pure head direction cells remained stable during both the introduction and the translation of the embedded space (Fig. 2, *B* and *C*). Differences in Rayleigh vector length of head direction in comparison to the open field condition were centered on zero, showing no bias toward an increase or decrease of head directional coding (Fig. 2*D*). Head direction cells also maintained their angular preference. This is obvious from the cumulative distribution of angle differences between home cage condition and open field condition, which is narrowly centered at 0° (Fig. 2*E*). Overall, there was a strong correlation between the head directional tuning curves with and without the home cage (Fig. 2*F*). The head directionality of conjunctive grid cells was also unaffected by the presence of the embedded space (Fig. 2, *G* and *H*), in both angle preference (Fig. 2*I*) and vector length [not shown, Kolmogorov–Smirnov (KS) normality test, *P* = 0.036, signed-rank test, *P* = 0.856, *N* = 39]. We further analyzed for differences of head directional encoding in head direction cells between inside and outside of the home cage (Fig. 2, *I* and *J*). Figure 2*I* shows the activity of a head direction cell inside and outside of the home cage. The cell has the same tuning properties inside and outside (Fig. 2*I*); this was observed in the population for both the home cage center and home cage rotated and translocated conditions (Fig. 2*J*). All in all we conclude that the presence of the embedded space, its translation, and relocation do not affect head direction tuning properties.

#### Absence of explicit egocentric home bearing cells.

**Fig. 3.**
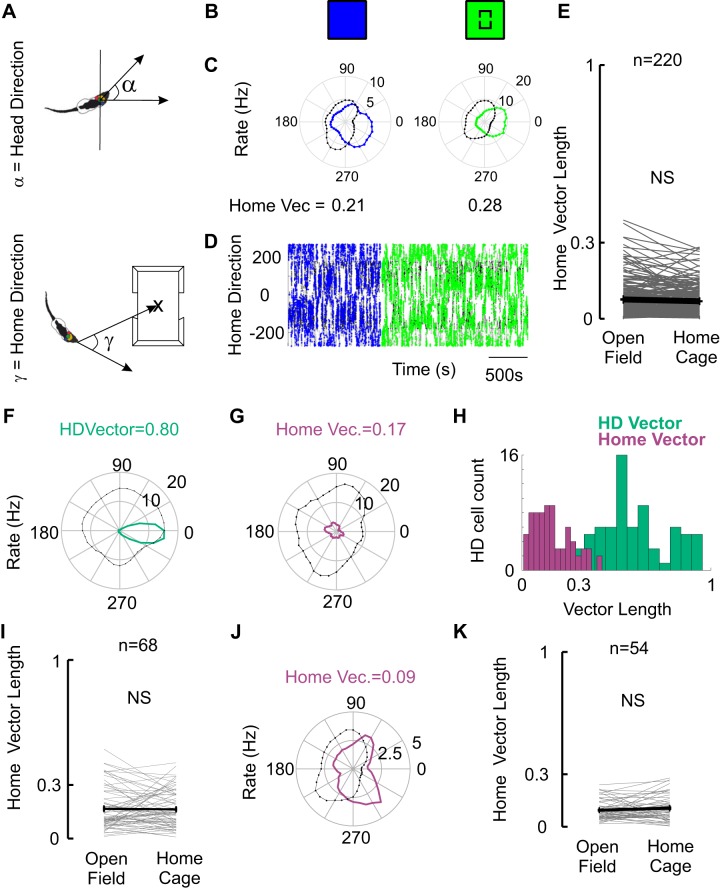
The home cage does not induce egocentric home bearing discharges. *A*, *top*: representation of head direction angle. *Bottom*: representation of home direction angle. Note that in this case corresponds to the angle between the head direction vector and the vector pointing from the head of the animal to the center of the home (x). Hence, the animal facing the home retrieves a value of 0 and the animal running away from the home results in a value of 180 or −180. *B*: schematic of the conditions depicted for a nongrid and non-head direction cell; open field (blue), home center (green). *C*: polar plots of home direction rates (blue and green according to *B*, showing no clear home direction vectors). *D*: plot of the home directionality of the spikes for this cell shows no clear home direction preference. *E*: distribution of fictive home bearing vector lengths in the open field (calculated to the center of the arena) and the corresponding vector lengths once the home cage is placed at the center of the arena. Mean and SE are depicted in black showing no significant increase in vector length. Kolmogorov–Smirnov (KS) normality, *P* = 0.0037, signed-rank test, *P* = 0.21, *N* = 220. All cells not classified as grid cells or head direction cells). NS, not significant. *F*: the head direction vector (HDVector) of one head direction cell. *G*: note the lack of clear home bearing tuning curve for the same cell as in *F*. *H*: the distribution for all pure head direction (HD) cells of home bearing vector lengths is much smaller than for head direction. *I*: home bearing vector lengths of pure head direction cells did not change with the presence of the home. KS normality, *P* = 0.367, *t* test, *P* = 0.560, *N* = 68. Gray lines show individual cells. Means are depicted in black. *J*: home direction polar plot for a grid cell, showing lack of home bearing encoding. *K*: home bearing vector lengths for all grid cells are very low and did not change with the presence of the home. KS normality, *P* = 0.780, *t* test, *P* = 0.250, *N* = 54. Gray lines show individual cells. Means are depicted in black.

As a second step, we assessed whether the embedded space could induce an explicit egocentric home bearing representation, i.e., neural discharges tuned to the direction of the home cage (Fig. 3). We therefore computed egocentric bearing to the home (home direction) as schematized in Fig. 3*A*, where a value of 0 corresponds to when the animal is facing the home cage (Fig. 3*A*, *bottom*). We performed this computation fictively in the open field in absence of embedded home cage (Fig. 3*B*, *top*, relative to where the center of the arena) and relative to the real home cage (Fig. 3*B*, *top*). Differences between the results of these two computations could be indicative of a home bearing representation. In Fig. 3, *C* and *D* we show data from one of the nongrid and non-head direction cells with the strongest home direction tuning in terms of vector length. As shown in the polar rate plot (Fig. 3*C*) and in the time resolved distribution of spikes in the home direction space (Fig. 3*D*), even in this cell there is no strong home direction tuning (vector length = 0.28). We did not find exemplary neurons convincingly encoding egocentric heading direction toward the center of the arena, or egocentric home bearing cells. We computed egocentric home bearing for the general population of cells recorded stably in these two conditions [*n* = 342, divided into Fig. 3, *E* (nongrid, non-head direction), *I* (head direction cells), and *K* (grid cells)]. For nongrid, non-head direction cells we found that the distribution of Rayleigh vector for the resulting egocentric home direction firing rates remains mostly below a cut-off level used for similar variables like head directionality vector length (0.3 in our case; Fig. 3*E*). The introduction of the home cage did not result in an increased encoding of egocentric bearing. We then investigated egocentric home bearing tuning specifically in both grid cell and head direction populations recorded in MEC and PaS to test for a representation of the egocentric home in a conjunctive way. This analysis did not reveal any egocentric home bearing tuning. Figure 3, *F* and *G* shows an example corresponding to a head direction cell with a very strong head directional vector length (Fig. 3*F*) and a very weak home bearing vector (Fig. 3*G*). We compared egocentric home bearing vectors lengths and head direction vectors lengths of the head direction cell population and found a large difference in their distribution strongly favoring head directionality (Fig. 3*H*). Besides presenting very low egocentric bearing vectors toward the center of the arena in the open field session, the presence of the home cage did not affect the encoding of egocentric bearing vectors in the head direction cell population (Fig. 3*I*). Similarly, grid cells did not represent egocentric home bearing. For the grid cell population we found very short home bearing vector lengths (Fig. 3*J*) and no effect of the introduction of the home cage in the environment (Fig. 3*K*). These data indicate that there is no egocentric bearing tuning toward the center of the embedded space in the global population of entorhinal and parasubicular cells here assessed, including grid cells, head direction cells, and nonclassified cells (Rest). However, we wondered whether such egocentric bearing encoding exists with respect to other regions of the home cage, for example its doors, or to other places in the arena. We complemented our analysis with a multipoint vector analysis.

#### Weak egocentric bearing representation from a multipoint perspective.

**Fig. 4.**
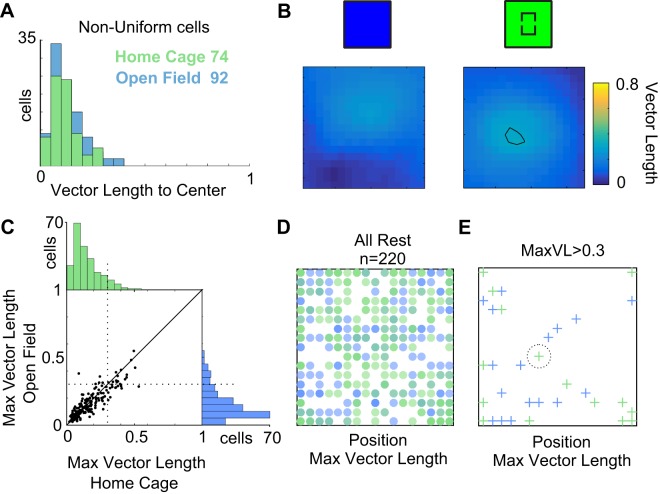
Weak egocentric bearing representation. *A*: distribution of egocentric bearing vector lengths with respect to the center of the arena for cells with significantly nonuniform egocentric home direction tuning curves. Only 4 significantly nonuniformly tuned cells are above the 0.3 cutoff for the open field condition (blue), 0 cells for the home center condition (green). *B*: multi-reference-point analysis for egocentric bearing direction. Egocentric bearing direction vector length maps for the cell in Fig. 3, in two conditions. We calculated egocentric bearing tuning vector length for each bin of space and computed the maximum vector length (MaxVL). *C*: distribution of maximum vector length values for all Rest cells [cells not classified as head direction cells, pure grid cells, or conjunctive grid cells (see text); *n* = 220]. Distributions show very few cells beyond cutoff of 0.3 vector length, even considering it is maximizing its module. *D*: position of reference point for MaxVL for all Rest cells (*n* = 220) in both conditions (blue: open field, green: home center). It can be noted that there is no apparent systematic bias in the location of the reference point, or a systematic bias for the center of the arena induced by the home. *E*: same plot as *D* for cells with MaxVL > 0.3 cutoff. The only cell with an egocentric bearing direction bias toward the position of the home is our example cell in *B* and in Fig. 3.

We assessed for egocentric bearing encoding from a possible multipoint perspective as has been described by other groups (LaChance et al. 2019; Wang et al. 2018), instead of just focused on the center of the home cage and the center of the arena. To compare the distributions, first we conducted a significance test for egocentric bearing tuning curves for the Rest population toward the center of the arena, by shuffling the spikes and calculating whether the tuning curves are significantly nonuniform (Fig. 4*A*). Few cells were significantly nonuniform and had an ample distribution of egocentric bearing vector lengths toward the center of the arena or the home cage; however, only four cells in the open field condition passed our head directional cutoff of 0.3 vector length (Fig. 4*A*). This implies that encoding toward the center of the arena or the home is at best very weak. Calculating the vector length of egocentric bearing to multiple points in the arena could lead to finding higher encoding of egocentric variables.

We next calculated the vector lengths of tuning curves for egocentric bearing toward a 17 × 17 lattice covering the environment. That gave us a map of vector lengths with reference to locations in space. Figure 4*B* shows the example cell in Fig. 3, *C* and *D* after following this analysis; it shows two 17 × 17 heat maps of egocentric bearing vector length in the open field and home cage center conditions. This cell shows a slight increase in vector length that goes barely beyond the 0.3 cutoff for a region close to the home. However, this was the only example of such behavior and not representative. Overall, we found the distribution of egocentric bearing vector lengths to remain very weak. For each cell of the Rest population we calculated the maximum vector length (MaxVL), the maximum of the multipoint vector length heat map. Figure 4*C* shows the distribution of MaxVL for cells in both conditions. The distributions of MaxVL found in our population of cells shows very low values, with few examples beyond cutoff (Fig. 4*C*), and fits well with the MaxVL distributions reported by Wang and collaborators for MEC (Wang et al. 2018) showing lack of egocentric bearing encoding. More importantly for our experiment, we do not find that the introduction of the home cage (green condition) increases significantly the MaxVL of the cells (Fig. 4*C*, comparison). We also checked whether the introduction of the home cage generated an apparent concentration of the positions for which the cells have a MaxVL, but we did not find an apparent effect considering all the cells (Fig. 4*D*), or considering the cells above cutoff (Fig. 4*E*). Only the example cell (demarcated), stands out as the only cell in 220 for which the inclusion of the home resulted in an above cutoff MaxVL close to the position of the home cage. Overall, we find that egocentric encoding in the PaS and MEC is weak when compared with head directional encoding, even from a multipoint perspective, and is not affected by the presence of the home.

#### Globally stable grid cells translocate single fields toward the embedded home.

**Fig. 5.**
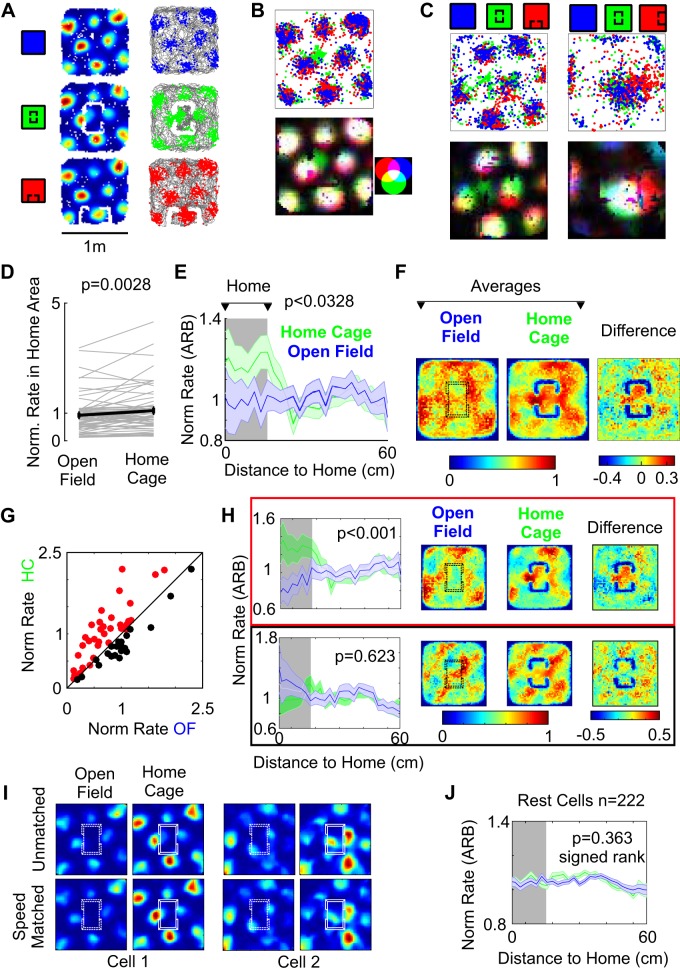
Single firing fields of grid cells shift toward home cage location. *A*: grid cell under 3 conditions: open field, home center, and home moved (blue, green, red respectively). *Left*: normalized rate maps. *Right*: spikes (RGB) superimposed on the rat trajectory (gray). *B*: composite plot of the spike positions for the three conditions of the cell in *A*. Note the change in positions for home center spikes. *Bottom*: composite rate map. Each rate map is normalized and assigned the corresponding channel in an RGB image. Side panel demonstrates the color compositions for the different RGB mixtures [cell recorded in dorsal medial the medial entorhinal cortex (MEC)]. *C*: two parasubicular grid cells, under the same 3 conditions as in *B*. Note how single fields move toward the position of the home. *D*: normalized firing rate increases in a region of the arena corresponding to the location of the home in comparison to the same area in the open field condition. ARB, arbitrary units. Kolmogorov–Smirnov normality, *P* = 0.280, *t* test, *P* = 0.0029, *N* = 51. Gray lines are individual cells. Mean and SE are depicted in black. *E*: the increase in normalized rate is evident in the Euclidian profile to the center of the home. Solid line corresponds to mean normalized rate, and shaded area correspond to the SE. Green: home center; blue: open field (*t* test for shaded area). *F*: the same effect is evident in the spatial averages of the peak normalized rates. Spatial averages of peak normalized rate maps for all cells in the open field (*left*) and home center (*middle*). On the right the difference between these two average maps is shown. *G*: brute force split between cells upmodulating their normalized firing rate in the home area (red, *n* = 32) and not-upmodulating (black, *n* = 19). *H*, *top*: same scheme as *F* for upmodulating cells from *G*, with the inclusion of the Euclidian profile to the left. *Bottom*: same scheme for downmodulating cells from *G*, with the inclusion of the Euclidian profile to the left (*t* test for shaded area). *I*: speed matching control of grid cell shifts. Two exemplary cells comparing unmatched and speed matched between home cage and open field conditions. Speed matching did not alter substantially the changes in grid pattern. *J*: lack of increase in normalized rate of Rest cells [cells not classified as head direction cells, pure grid cells, or conjunctive grid cells (see text)] in the Euclidian profile to the center of the home. Solid line corresponds to mean normalized rate, and shaded area correspond to the SE. Green: home center (HC); blue: open field (OF) (signed-rank test for shaded area).

Third, we assessed how the embedded home affects positional signals and in particular grid cell discharges (Fig. 5). As already implied by the stability of the head direction system to home cage insertion, the animal must remain familiar with the global environment. In line with the lack of effect on head direction cells, grid cells did not globally remap because of home cage insertion. Global average firing rates were not altered by the presence of the home (KS normality, *P* = 0.384, *t* test, *P* = 0.541, *N* = 51). We observed, however, that a proportion (60%) of grid cells recorded in both the PaS and the medial MEC in the presence of the home cage did alter their discharge patterns. Figure 5, *A* and *B* shows a grid cell recorded in the MEC, the normalized rate maps for the cell (with a 5 cm/s speed threshold to exclude stasis), and the trajectories and position of the animal when the cell fired (in corresponding color code). The introduction of the home cage in the center of the arena retained the global representation but resulted in a local shift of a single firing field toward the embedded home. It can be clearly observed that the central field in the green center condition is shifted toward the location of the home cage. This is visible both at the level of spike positions (Fig. 5*B*, *top*) where the green spikes are clearly shifted in position or in Fig. 5*B*, *bottom*, where a composite normalized rate map using the RGB color scheme also clearly depicts the displacement of the central grid field in the green condition. Figure 5*C* presents two further examples of grid cells modifying their activity by translocating fields toward the location of the home cage. Movements of grid field centers were not large (median = 9.26 cm, SD = 3.2, *n* = 15). However, shift of spikes may contribute further than the translocation of the center. We wondered whether these shifts affect the rates of grid cells inside the embedded home. To be able to compare grid cells with distinct firing rates and phases, we looked at rates of individual cells normalized to the average rate of the cell. If we compare normalized rates of the grid cells inside the embedded space, with the mean normalized rates in the equivalent area of space during the open field session, we note a significant increase in these rates in the population (Fig. 5*D*). This change is also evident in the change in the profile of mean normalized rates with the Euclidian distance to the center of the embedded home (Fig. 5*E*). Spatially averaging peak normalized rate maps of all grid cells in the open field and in the centered condition shows a local spatial increase in the firing rate, which can be further visualized by calculating the difference between the averages (Fig. 5*F*, *right*). Equal results can be obtained by normalization of spatial maps to the mean (results not shown). However, not all grid cells increased their normalized rate inside the embedded space; a smaller fraction did not upmodulate its firing rate (Fig. 5*G*). We used the unity line as an ad hoc classification to disentangle possible differences due to the original configuration of the grid. Once we separated these two populations and performed the spatial averages and Euclidean average (Fig. 5*H*), it became clear that positively modulating cells contribute to the overall effect. This result is of course tautological, but we observed in addition that the average of these cells in the open field session had a low rate in the area where the home cage was to be placed. These observations indicate that introducing the embedded space boosted firing in cells, via node translocation, that initially had no grid firing node in the center of the arena (Fig. 5*H*, *top*). On the other hand, unchanged cells tend to have an original field in the center of the arena, where the home cage will be placed (Fig. 5*H*, *bottom*). Collectively, these observations show that the grid cells, which do not have a firing node in the embedded space location, locally alter their firing patterns. Specifically it appears that a firing nodes close to the home cage get “sucked” into it. This effect persisted after correcting for differences in speed between sessions. We used the speed matching approach used by Butler et al. (2019), to confirm that local changes in grid cell activity are not due to differences in running behavior (Fig. 5*I*). We did not, however, find systematic increases in normalized rate inside the home for the population classified as Rest (Fig. 5*J*).

**Fig. 6.**
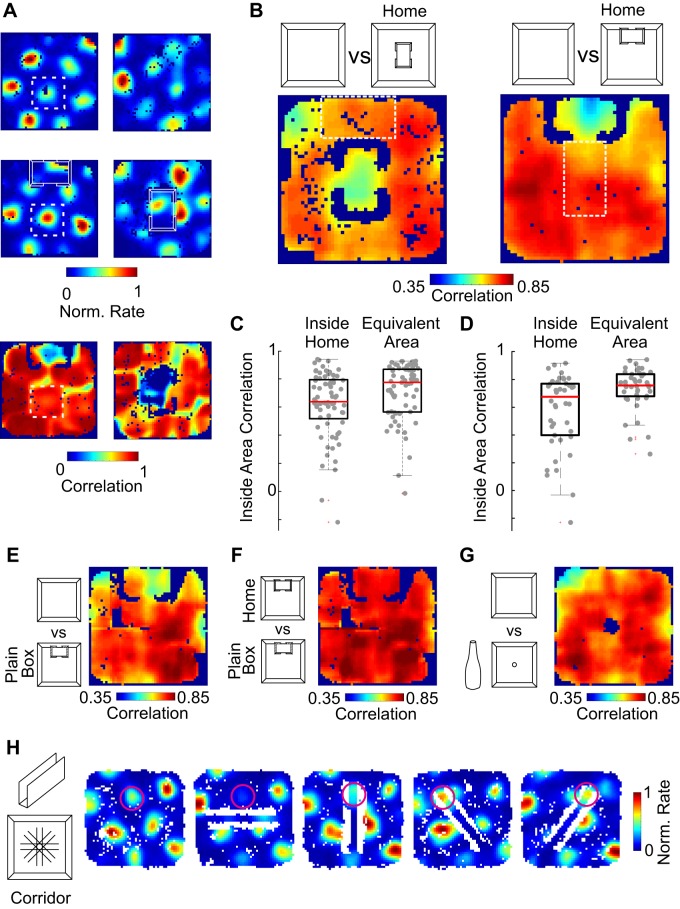
Effect of home on grid cell population is driven by geometry. *A*: sliding window correlation analysis between conditions. *Left*: example of peak normalized rate maps of a grid cell in the open field (*top row*) and home north (*middle*) condition, and the corresponding sliding window correlation between these two (*bottom*). *Right*: same as *left* but for another cell compared with the home center condition. *B*: sliding window correlation analysis for all grid cells in both conditions. Note the lower average local correlation in the position of the home for both cases. *C*: inside embedded space correlation for the home cage center condition, and for an equivalent area (white dashed box in *B*). The presence of the home produces a lower correlation in comparison to an equivalent area that remains unchanged (signed-rank test, *P* < 0.001, *N* = 69). Black box corresponds to interquartile range, and the horizontal red line corresponds to the median. *D*: inside embedded space correlation for the home cage moved condition, and for an equivalent area (white dashed box in *B*). The presence of the home produces a lower correlation in comparison to an equivalent area that remains unchanged (signed-rank test, *P* < 0.001, *N* = 42). *E*: sliding window correlation analysis comparing the home versus a cardboard box with similar dimensions, showing that the effect is related to geometry not valence of the home. *Left*: the comparison between the open field and the cardboard box is shown. *F*: high correlation comparison between the home and the cardboard box. *G*: sliding window correlation analysis between the open field and a tall object in the center of the arena did not produce low correlations near the object. *H*: the presence of a linear corridor in the arena in different angles produced shifts of grid fields in some of the conditions further demonstrating the strong effect of internal geometry. The moving field is highlighted in pink.

To quantify the local grid cell changes we performed a sliding window correlation analysis (Wernle et al. 2018) between the peak normalized spatial rate maps in the open field and home cage conditions. For any given pixel in the arena we selected a surrounding squared region (of dimension similar to the cells average grid spacing). This region matches spatially for both conditions (Fig. 6*A*). For each region the cell’s firing rates were correlated between open field and home cage conditions. The result is a heat-map of local correlation values between the different conditions. In this example, the presence of the home cage reduced locally the correlation of the grid (Fig. 6*A*, *bottom*). We averaged these correlation maps for all grid cells and found that these show a mean local decrease in correlation corresponding to the location of the home cage in the center (Fig. 6*B*, *left*). Notably, the decrease in mean correlation follows the embedded home, when we moved it to the side of the arena (Fig. 6*B*, *right*). The decorrelation of grid cell activity appears to be local and related to the position of the embedded space in the global environment. This analysis reinforces the idea that presence of the home cage resulted in a change in the local activity of the grid while not affecting the global encoding of space. We quantified in the population of grid cells the mean correlation inside the home cage and compared it with an equivalent area outside of the home (Fig. 6, *C* and *D*). For both the home center (Fig. 6*C*) and home moved (Fig. 6*D*) conditions, we find that the mean correlation of the pixels inside the home area is lower than an equivalent area outside of the arena (depicted as dashed lines in Fig. 6*B*).

We were curious whether this effect might be driven or intensified by the positive valence and familiarity of the home cage utilized as an embedded space or whether grid cells are just encoding for the changes in local geometry introduced. To disentangle this we performed additional controls with a cardboard box with equal dimensions to the home cage, yet without both the familiarity and the social valence of the home cage. We found, however, that the correlated activity of grid cells in the presence of the box drove a similar local change with respect to the open field as had the home cage (Fig. 6*E*). Convincingly, activity in the presence of the box correlated strongly with the activity in the presence of the home cage (Fig. 6*F*). Hence, we believe that the effect seen of grid cells is unrelated to home saliency but related to the geometrical modification in the internal structure of the environment. This could be related to just the presence of an “object” in the environment. To dissect this further we tested whether tall objects that do not alter the space substantially have a similar local effect on grid cells. We found that the simple presence of an object does not decrease the correlation of grids around it (Fig. 6*G*). These results point toward the requirement of an embedded space in the environment that would considerably affect the trajectories available to the rat. We wondered whether single tall objects do not produce the same effect, perhaps because they minimally affect the availability of paths in the environment. Pursuing this idea further we presented the animals with new internal structures inside the environment. We used an open corridor of 70 × 10 cm, which we could place in the arena in different orientations. The presence of a corridor, a linear embedded space, inside an open field grossly changes the availability of paths in the same familiar environment. Our prediction would be that grid cells would be influenced by the position of this corridor and modify their firing fields to better represent this salient path. Introducing such corridor revealed big shifts in grid fields toward the corridor in some, but not all conditions (Fig. 6*H*, moving field highlighted in pink). This example shows a single field, pulled in two different directions in only two conditions, further demonstrating the effect of internal geometry in purely local grid cell activity.

#### Explicit behavioral use of the embedded home does not cause remapping.

**Fig. 7.**
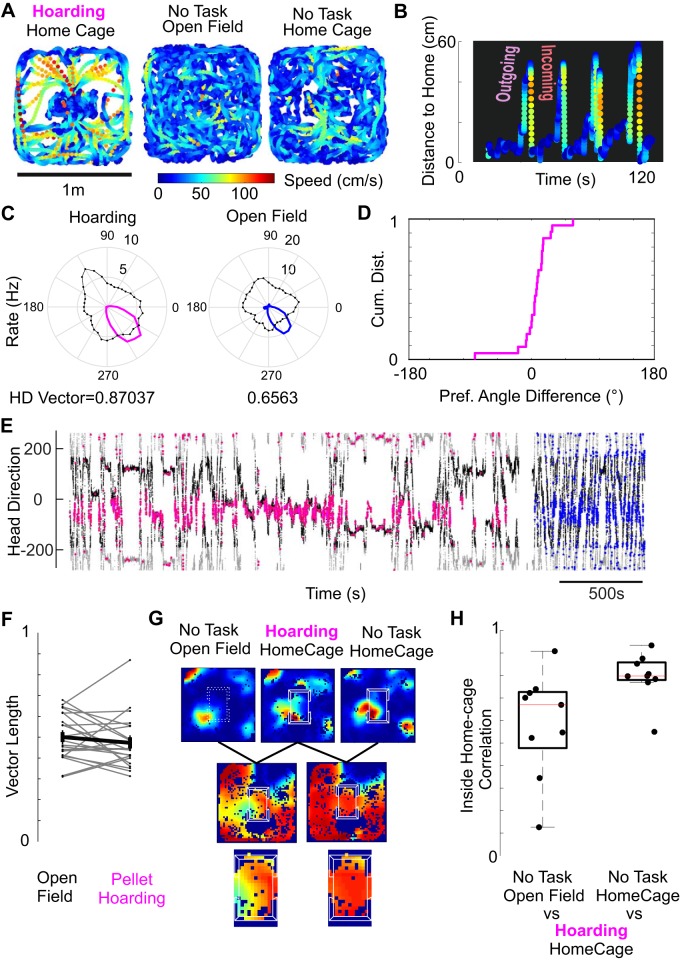
Head direction discharge and grid cell activity is not strongly altered by a pellet hoarding task. *A*, *left*: behavior of the rat during pellet hoarding task, presenting high speeds and centralized home basing behavior. *Middle*: behavior of the rat in the open field while foraging for chocolate treats. *Right*: behavior of the rat with the embedded home cage, but while foraging for chocolate treats, behavior closely resembles open field behavior. *B*: home cage and pellets produces homing behavior in the rat. Rats spend time in the home and have outgoing trips to find pellets and incoming trips to hoard them in the home. Incoming trips are faster than outgoing trips. *C*: head direction rate polar plots (rate in corresponding color, angular occupancy in black). *D*: cumulative frequency function of the distributions of differences between preferred angle in the open field and in the pellet hoarding conditions. Note that the inflection point is at 0 and the very steep slope. Kolmogorov–Smirnov (KS) normality test for angle difference, *P* = 0.6993). *E*: spikes of the head direction cell are clearly preferring a stable head direction. Black and gray, head direction of the animal in time (duplicated for visualization). Spikes are plotted on *top* in the corresponding color scheme. *F*: head direction Rayleigh vector lengths are not systematically affected by the presence of the home cage during the execution of the hoarding task. KS normality test, *P* = 0.0.851, signed-rank test, *P* = 0.426, *N* = 20). Gray lines are individual cells. Means are depicted in black. *G*: inside home cage correlation comparison. The hoarding/home cage condition is compared on one had to the no task/open field condition and on the other hand to the no task/home cage condition. Example correlation analysis shows a higher correlation inside the home cage between the two conditions with the embedded space present. *H*: inside home cage area correlation for both comparisons in *F*. Showing high correlation between both conditions including the embedded space and lower correlation between the hoarding session and the open field (signed-rank test, *P* = 0.0039, *N* = 9).

To assess whether the explicit use of the embedded space could differently affect head direction cells and grid cells, for a smaller population of cells we recorded while the rat was performing a pellet hoarding task. During pellet hoarding (Fig. 7, *A* and *B*; Wolfe 1939) rats forage for food pellets and perform high-speed return vectors toward their safe location. In our setting (Fig. 7*A*) rats foraged large food pellets in a 1-m arena in the presence of their home cage. Without specific prior training rats foraged these pellets and cached them in their home cage. The behavior was stereotypical, consisting of high speed return trips as previously described in the literature (Maaswinkel and Whishaw 1999; Winter et al. 2018) (Fig. 5*B*).

Running speed was visibly lower during exploratory trips away from their home (Fig. 5*B*) as has been described in the literature (Tchernichovski et al. 1998; Wallace et al. 2008; Winter et al. 2018), incoming trips consisted of higher speeds (KS normality, *P* = 0.747, *t* test, *P* < 0.001) than outgoing. Thus, in the presence of large food pellets the home cage in the arena setting greatly altered the rat’s locomotion patterns and divided them into irregular, slower exploratory outgoing trajectories and relatively straight, faster return trips to the home cage. These observations suggest that the home cage can induce homing behaviors, even in a scenario where the rat is well adapted to the global environment.

We used this behavior to probe whether the explicit use of the home cage now as a home base for hoarding had disruptive effect on head direction cells and grid cells. We compared the activity of head direction cells in the open field with the presence of the home cage during performance of the pellet hoarding task. We find that the task demands do not induce a remapping of head directionality (Fig. 7*C*): head direction cells remain encoding for the same head direction. We found that the maintenance of the head direction angle to be the norm across a population of head direction cells and conjunctive grid cells (*n* = 22) (Fig. 7*D*). Figure 7*E* shows the head direction angle variable plotted in time and the head direction the animal was in at the time of the spikes, to show the consistency of the firing activity over these two very different sessions, regardless of the clear differences in behavior. We also did not observe a systematic difference in head direction vector length between conditions (Fig. 7*F*). Overall, the performance of a task did not induce remapping of head directionality.

We next turned to the effect of the hoarding task on grid cell distortion. In a small population of cells (*n* = 9), we performed sliding window correlation analysis between the hoarding condition versus, on one hand, the home cage center condition in the absence of pellet hoarding and on the other hand against the open field (Fig. 7*G*). Due to sparse occupancy outside of the home, we looked at correlation of grids inside the home cage. Figure 7*G* shows that correlations inside the home cage area are higher for the comparison of hoarding-home cage than no task-hoarding. This is due to the fact that the grid is being distorted by the placing of the home cage alone; furthermore, explicit use of the embedded home for a pellet hoarding task did not overhaul grid cell activity inside the home. Overall, in these few cells we found a significant difference between these correlations (Fig. 7*H*), implying that grid cells are not differentially affected by the task.

## DISCUSSION

The home cage is a highly relevant location for the rat. Consistent with this idea the home cage was capable of inducing characteristics of natural homing behavior. Even though the PaS and the MEC are causally linked to the expression of spatial navigation, and contain a panoply of cells encoding variables linked to space and orientation, we did not find an explicit firing rate representation of a home bearing. Specifically, we did not find home bearing representation with the same quality of needlelike encoding as the goal direction cells found by Sarel et al. (2017) in the hippocampus of freely flying bats with respect to a homelike platform. Our result falls in line with recent work finding a dissociation between allocentric bearing encoding in the MEC versus egocentric bearing encoding in the LEC (Wang et al. 2018) and does not find sharp encoding of egocentric bearing direction in relation to the center of the arena or the embedded home as has been found by Wang and has recently been described in postrhinal cortex (LaChance et al. 2019). We find that, even considering multipoint egocentric bearing, the PaS and MEC do not seem to have strong encoding of egocentric bearing and are not affected in that regard by the introduction of the home cage. This also falls in line with recent work (Wang et al. 2018) and extends such results to the PaS.

We found that both head direction cells and the head directional component of conjunctive grid cells are not affected by the presence of the home cage. The preferred head direction is retained, even if the home is translated to one of the sides of the arena, breaking symmetry even further. This points to head direction cells and grid cells maintaining their encoding of global familiar environments, i.e., there is no remapping because of the insertion of the home. Head directionality is also not differential between inside and outside the embedded home, also implying global not local anchoring of head directional responses. The fact that the rat was never removed from the arena or disoriented between sessions probably explains the consistency of head directional encoding for the global environment and its subcompartments. Many of the traditional cue card rotation experiments where head direction realignment was found were performed in cue-deprived conditions and with a step of disorientation of the animal between sessions.

Even though earlier work had shown that grid cell macro crystalline hexagonal patterns break in complex shaped environments like the hairpin maze (Derdikman et al. 2009), and more recent studies have shown global distortions are possible when exposing the animal to irregular shaped environments or changing external boundaries (Krupic et al. 2015, 2018), we are just beginning to understand how the structure of the environment affects single firing fields and whether these distortions might contribute to encoding more intricate environments.

In a similar manner a study by Wernle and collaborators shows a much faster change in the grid pattern of cells encoding two environments separated by a wall, before and after said wall is removed (Wernle et al. 2018). Grid cells showed a tendency toward a more global representation by stitching up the previous two global grid patterns, into a metaglobal grid pattern. This intriguing study points to a strong role of borders in the establishment of the grid pattern. However, in this case it is hard to disentangle what might be the single contribution of local structures, since the overall global environment is changed in shape and size.

Part of our contribution lies in testing what are the micro level effects of an embedded space as part of the environment (in our case the rat’s home cage) on grid fields of well learnt, familiar environments without disturbing the global environment itself. In line with the head direction, grid cells did not globally remap with the presence of the embedded home. In all cases they retained their previous phase, orientation, and spacing, as evidenced by the high correlation of rate maps between conditions. However, we did find that grid cells are far from being a perfect crystal projected on to the arena. We observed shifts of local firing fields toward the position of the embedded space. This could very well be a translation toward the center of the space itself, or toward the entryways. Our paradigm cannot resolve this question unequivocally. More precise experiments could unravel the precise role of a doorway in these shifts.

This phenomenon manifested itself robustly by a local decrease in correlation of the rate maps of the grid cells inside the home cage, while global correlation of the grids is preserved. This low correlation could also be a consequence of the embedded nature of the home and could potentially be recruiting simultaneously two different spatial reference frames and simultaneous grid maps. Observing such phenomenon would require embedding spaces larger than the home cage used. Work on CA1 place cells has shown that subspaces inside a larger space are capable of recruiting different hippocampal maps (Fenton et al. 2008); however, in such cases, rats were not allowed to transverse outside of the subspace cylinder into the outer arena. Studies on place cells under transversed multicompartmental environments have shown place field repetition on parallel indistinguishable arenas (i.e., recruitment of the same map) and that orientation of the multicompartmental environment in a radial fashion allows for recruitment of independent maps and spatial learning (Grieves et al. 2016). These results point to a role of correct integration of head direction for adequate spatial navigation in multicompartmental environments (Harland et al. 2017).

Our results fall in line with recent work showing that single grid fields moved to reward locations (Boccara et al. 2019), implying a local plasticity of node locations. However, we did not see any global remapping (Butler et al. 2019) of head direction cells or grid cells due to the simple presence of the home, even though we do see an increase in the home location representation because of field convergence. We did not see any global remapping related to the execution of a task either; however, this might be due to the fact *1*) that rats are not executing a memory-related task or *2*) that we didn’t enforce a pairing of the task with a completely new environment (Butler et al. 2019). Butler and colleagues paired a memory task with a different global environment, resulting in head direction and grid remapping and overrepresentation of the rewarded location. We wonder whether, if their memory task was executed in the same familiar environment, the cells would globally remap while performing the task, or whether more subtle effects of single moving fields like those reported by Boccara and colleagues and our study would be observed. Task-related remapping has been observed in clear ways in the hippocampus where context related hippocampal activity is different even in the same environment in an alternate T-maze task (Ainge et al. 2007; Wood et al. 2000).

The observed relocation of grid fields due to embedded spaces in the environment may have a critical role in vector-based navigation and route planning. The most recent interpretation of the role of grid cells in spatial navigation relates to the possibility of using grid populations to directly calculate the shortest path between two points (Banino et al. 2018; Kubie and Fenton 2009). Changes in the internal structure of the environment restructure the availability of paths in the environment. Some paths need to be better represented, while some others are now impossible. The hexagonal grid cell in the open field might be the optimal way of encoding all possible segments, which for example includes all possible directions. As has been described, when the same cells are recorded in a hairpin maze, grid cells become linearized inside each transect and repeating for different pins of the maze and at the same time becomes dependent on the direction of travel (Derdikman et al. 2009). This restructuring of the grids is in line with the restructuring of possible paths in the arena; the only vectors that can be calculated are ones going back and forth through each hairpin. When rats were trained to perform a zigzag in an open field environment, behaviorally mimicking the hairpin-like behavior, the gridness of the cells was once again present, given that all the possible paths were again available (Derdikman et al. 2009). A similar reasoning can be applied to recent results showing grid cells preferentially encoding memorized goal locations (Boccara et al. 2019; Butler et al. 2019). A goal location changes the affordance of space and the type of paths in space that are to be encoded. In their case, reward is the driving force for grid cell distortion, while here we show that embedded geometry has a similar effect.

The shifts found for grid cells together with the upmodulation of firing rates in the home point toward an increased overlap in the grid population firing in the position of the home. This could allow for a more resolved encoding of that area of the environment. Our results are in line with recent work showing the adaptability of grid cells to changes to their global environment (Krupic et al. 2018; Wernle et al. 2018). In addition, we have shown that grid cells also flexibly encode local internal changes in the environment, pointing toward their role in encoding more naturalistic environments and suggesting a clear hypothesis toward their role in allowing vectorial navigation in complex environments.

While our study confirms on a behavioral level that the home cage is a unique location of rats, we have not been able to decipher a neural signature of what makes home a special place. Our hoarding behavior in Fig. 7 shows that animals spontaneously use their home cage for naturalistic home-driven behaviors, like hoarding. On the other hand there is the question of whether the home cage matters to the neurons in MEC and PaS. This we report clearly as a negative finding. A lack of home-specific-related activity in MEC and PaS does not generalize to the brain or to the importance of the home cage for the animal’s behavior. We have not found a home bearing representation, or an upheaval of head direction cells and grid cells due to the saliency of the home cage. We wonder whether such a “neural home signature” exists in the corticohippocampal system or whether subcortical circuits provide the animal with this information.

## GRANTS

This study was funded by the Einsteinstiftung (Einstein Stiftung Berlin project “Dynamics of electronically coupled neuronal networks” A-2016-350).

## DISCLOSURES

No conflicts of interest, financial or otherwise, are declared by the authors.

## AUTHOR CONTRIBUTIONS

J.I.S.-S. and M.B. conceived and designed research; J.I.S.-S. performed experiments; J.I.S.-S. analyzed data; J.I.S.-S. and M.B. interpreted results of experiments; J.I.S.-S. and M.B. prepared figures; J.I.S.-S. and M.B. drafted manuscript; J.I.S.-S. and M.B. edited and revised manuscript; J.I.S.-S. and M.B. approved final version of manuscript.
